# The influence of timing of surgery in the outcome of spinal cord injury without radiographic abnormality (SCIWORA)

**DOI:** 10.1186/s13018-020-01743-1

**Published:** 2020-06-16

**Authors:** Can Qi, Hehuan Xia, Dechao Miao, Xingui Wang, Zengyan Li

**Affiliations:** 1 Department of Orthopedics, The Third Affiliated Hospital of Hebei Medical University, 139 Ziqiang Road, Shijiazhuang, 050051 Hebei China; 2 Department of Spinal Surgery, The Third Affiliated Hospital of Hebei Medical University, Shijiazhuang, Hebei China

**Keywords:** Spinal cord injury, Clinical value, Duration from injury to surgery, Surgical treatment, SCIWORA, Spinal cord concussion

## Abstract

**Background:**

Spinal cord injury without radiographic abnormality (SCIWORA) is a rare traumatic myelopathy. Although surgery is one of the most important treatments, the surgery for SCIWORA is controversial, especially the time of surgery is a topic of controversy. Here, we investigate the effects of difference in duration from injury to surgery on the outcome of SCIWORA.

**Methods:**

This retrospective study was performed in all patients with spinal cord injury admitted to the Third Affiliated Hospital of Hebei Medical University from January 2013 to April 2017. Fifty-seven patients who met the study requirements were divided into 3 groups according to the duration from injury to surgery. Group A (surgery within 3 days of injury) had 18 patients, group B (surgery within 3–7 days) had 18 patients, and group C (surgery later than 7 days) had 21 patients. All the groups were compared with Mann–Whitney *U* test; the functional improvement of spinal cord was compared and analyzed using the ASIA sports score and ASIA Impairment Scale (AIS).

**Results:**

There was a significant improvement in the long-term AIS (final follow-up) in all the 3 groups compared to before surgery. The final follow-up recovery rate of group C was worse than group A and group B. The curative effect of operation within 7 days was significantly better than the surgery done 7 days later. The recovery rate of group C was worse than group A and B. The ASIA sports score showed that recovery was quicker in the early stage and slow in the later stage.

**Conclusions:**

The optimal schedule of surgical treatment was 3–7 days after injury, which can significantly improve the short-term and long-term follow-up effects. Longer the time to surgery from the time of injury, the worse was the prognosis.

## Introduction

Spinal cord injury without radiographic abnormality (SCIWORA) is a rare traumatic myelopathy without any evidence of vertebral fracture or malalignment on plain radiographs or computed tomography (CT). Pang et al. [1] SCIWORA was first reported in children in 1989. However, a large number of studies later discovered the existence of SCIWORA in adults, and Hirsh et al. [2] applied the definition of SCIWORA to adults, who reported cases of thoracic spinal cord injuries (SCIs) without reflex abnormalities for the first time, since then, reports of SCIWORA in adults have increased. SCIWORA in adults mainly affected the cervical spine and was associated with a wide range of movements in the cervical spine [3], among which C3-4 and C5-6 were vulnerable segments [4]. The most common injury mechanisms of SCIWORA are hyperextension, flexion, and distraction of the head [5], characterized by short damaged segments, mostly incomplete SCI, and severe paresthesia below the level of SCI [6]. Most patients have incomplete SCI, and the prognosis is poor upon the occurrence of complete SCI. Nonsurgical treatments such as steroid therapy, fixation, and most importantly, avoiding activities that may increase the risk of secondary injury are the primary treatment options for SCIWORA patients. Despite the role of surgical treatment in SCIWORA being controversial, surgical treatments such as decompression and fusion should be considered for patients with clinical and MRI evidence of persistent compression and instability of the spine. The primary surgical method is anterior approach, and the common surgical methods are anterior cervical discectomy with interbody fusion (ACDF) and anterior cervical corpectomy and fusion (ACCF).

Currently, the primary principle of the treatment for SCIWORA is to relieve spinal cord compression and to stabilize cervical spine sequence. However, in some patients, cervical trauma leads to spinal concussion, which is very similar to SCIWORA in clinical manifestations making it difficult to distinguish between the 2 indications at present. Therefore, the optimal duration from the diagnosis of injury to surgery of SCIWORA is controversial, excluding spinal concussion. Meanwhile, the mechanism of injury is also a topic of debate. The purpose of this study was to investigate the influence of difference in duration from injury to surgery on the postoperative efficacy of SCIWORA in adults, to provide a basis for the selection of surgical timing for such injuries in the future.

## Materials and methods

The American Spinal Injury Association Impairment Scale (ASIA) grading system [7] was used in this study, with complete injury (grade A) to normal (grade E).

Data of all adult patients with SCI admitted in the Third Affiliated Hospital of Hebei Medical University from January 2013 to April 2017 were retrospectively studied. During the 4-year follow-up study period, 506 patients admitted in the hospital were identified to have SCI based on the International Classification of Diseases, Ninth Revision codes. Patients with (a) radiological evidence of trauma (fractures, dislocations, or subluxations), (b) presence of SCI at the thoracic or lumbar region, (c) pathological lesions in the spine, and (d) trauma with brain/chest/abdominal injury were excluded from the study. Patients with degenerative changes were not excluded. Fifty-seven patients who met the inclusion criteria were included in the study.

The patients were divided into 3 groups according to the duration from injury to surgery (Table 1). Surgery done within 3 days was categorized as group A, which had 18 patients, of which 12 were males and 6 were females, age ranging from 24 to 74 years old (52.44 ± 2.76). Neurological examination was performed every 2 h within 24 h of admission, and neck collar was worn until surgery. Anterior cervical decompression and bone grafting and internal fixation (ACCF or ACDF) were performed within 3 days. Group B included patients who underwent surgery within 3–7 days, which had 18 patients, 14 males and 4 females, age ranging from 28 to 64 years old (47.89 ± 2.55). Surgical treatment was performed 3–7 days after injury on the same basis. Group C had patients who underwent surgery more than 7 days after the injury occurred which included 21 patients, 18 males and 3 females, age ranging from 23 to 67 years old (47.71 ± 2.80). In this group, 20 patients underwent surgery within 30 days, with only one exception, 60 days after the injury. Surgery was performed 7 days after the injury in the same procedure.

**Table 1:** Statistical analyses of relevant factors in the improvement rate of the ASIA sports score and AIS

	Sex (M/F)	Age (year)	MRI abnormalities (Ex/Ex-in)	Duration from injury to surgery (day)
Group A	12/6	52.44 ± 11.72	14/4	2.22 ± 0.73
Group B	14/4	47.89 ± 10.80	15/3	5.78 ± 0.88
Group C	18/3	47.71 ± 12.85	15/6	14.95 ± 11.40

*ASIA* American Spinal Injury Association, *AIS* ASIA Impairment Scale, *Ex* extraneural, *Ex-in* extra and intraneural

Continuous data are presented in mean ± SD

A preoperative and postoperative grading was carried out on all patients and were graded according to the AIS, and their motor function was evaluated according to the ASIA sports score. The score was mainly based on the score of motor function of the 10 pairs of key muscles in the upper and lower limbs, 5 points for each muscle, and 100 points in total. Recovery rate = (ASIA sports score at follow-up-preoperative ASIA sports score)/(100 − preoperative ASIA sports score) × 100%.

The SPSS version 21.0 statistical software (IBM, Armonk, NY, USA) was used for statistical analysis. Kolmgorov–Smirnov test was used to verify whether the data conformed to the normal distribution. Data were recorded as median (25 to 75% percentiles). Mann–Whitney *U* test was used to compare the recovery rate among the 3 groups. *P* < 0.05 was considered statistically significant.

## Results

### Patient population and disposition

The 57 patients who met the inclusion criteria were characterized as 37 cases of fall injury (64.91%), 15 cases of car accident injury (26.32%), 4 cases of heavy object smashing injury (7.02%), and 1 case of traction injury (1.75%). In this study, 8 patients had complete SCI and 49 patients had incomplete SCI. The follow-up period was from month 22 to 73 until February 2019, with an average of 51 months.

In this study, 8 cases were classified as grade A, 8 cases as grade B, 23 cases as grade C, and 18 cases as grade D. All patients underwent cervical X-ray, CT, and MRI imaging before surgery. All 57 patients had abnormal MRI scan results; of these, 44 patients (77.19%) had extraneural and 13 patients (22.81%) had combined extra and intraneural MRI abnormalities.

During the 4-year (2013 to 2017) period of this study, 506 admitted patients were identified to have SCI. Of these, 58 and 134 patients were excluded as they had SCIs at C1-2 and thoracic or lumbar region, respectively. Apart from this, 106 patients were excluded from the analysis because of the association with craniocerebral or thoracoabdominal injuries; of the remaining 151 patients, only 127 received MRI. Sixty-three patients diagnosed with fractures during CT scan were excluded, and therefore, the data analysis included only the remaining 64 patients who were diagnosed with SCIWORA. Finally, in this study, 3 patients missed to follow up and 4 patients were excluded because of posterior cervical spine surgery. The follow-up time ranged from 22 to 73 months, with an average of 51 months. Notably, none of the patients treated in this study had developed serious complications.

### Improvements in long-term follow-up

In the final follow-up, the long-term AIS of the 3 groups was compared to the AIS before surgery and the improvements were noted by at least one grade (Table 2).

**Table 2:** AIS at admission, 6-month, and final follow-up

At admission	A	B	C	D	E
Six-month follow-up					
A	8				
B		1	7		
C			2	21	
D				18	
Final follow-up					
A	3	5			
B			3	5	
C				21	2
D				4	14

*AIS* ASIA Impairment Scale

The 6-month follow-up recovery rate of group C was lesser than group A (*Z* = − 2.663; *P* = 0.008) and group B (*Z* = − 2.099; *P* = 0.036), and the final follow-up recovery rate of group C was also lesser than group A (*Z* = − 2.953; *P* = 0.003) and group B (*Z* = − 2.693; *P* = 0.007) (Table 3). However, there was no significant difference in neurological recovery rate between group A and group B. The curative effect of the surgery within 7 days was significantly better than the surgery conducted after 7 days of injury.

**Table 3:** Preoperative, postoperative follow-up ASIA sports score, and postoperative follow-up recovery rate

Preop		6 months of follow-up	6 months of follow-up (%)	Final follow-up	Final follow-up (%)
A	75.00 (49.00–82.00)	91.50 (75.00–96.00)	66.10 (44.98–78.07)	98.00 (87.00–100.00)	89.59 (74.82–100.00)
B	72.50 (43.00–83.75)	91.00 (64.75–96.00)	67.95 (34.68–76.80)	98.00 (78.00–100.00)	89.90 (61.40–100.00)
C	72.00 (62.50–77.50)	84.00 (73.50–89.00)	41.94 (31.98–50.00)	90.00 (84.00–94.00)	66.67 (53.62–75.00)

*ASIA* American Spinal Injury Association

*Values are expressed as the median (interquartile range)

**Fig. 1:**
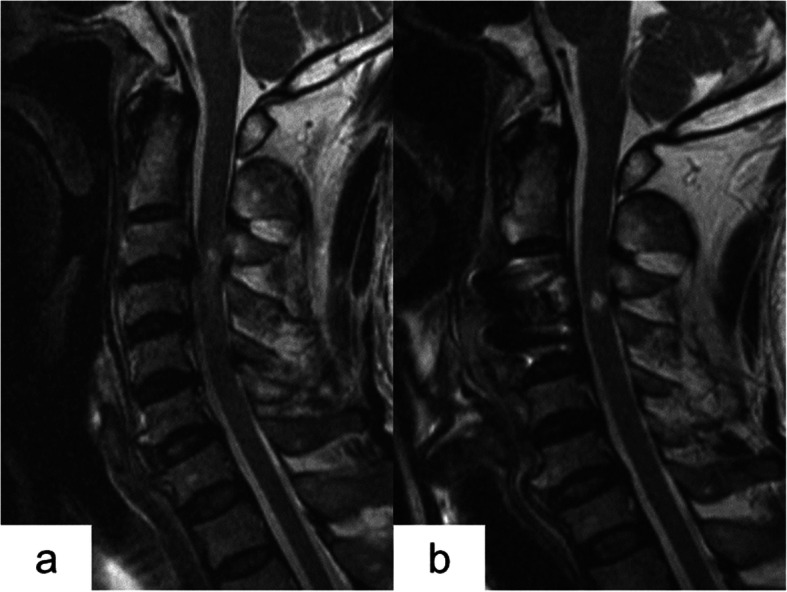
Sagittal magnetic resonance imaging (MRI). **a** Preoperative MRI showing C3-4 disc herniation and high signal in the spinal cord. **b** Postoperative spinal cord compression relieved, but MRI showing high signal range of the spinal cord still existing, and the signal becoming stronger

There were 2 patients in group A, whose neurological functions were found to be worse during the final follow-up than 1-year follow-up, and abnormal signals were still found in the spinal cord when re-examined in MRI (Fig. 1).

**Fig. 2:**
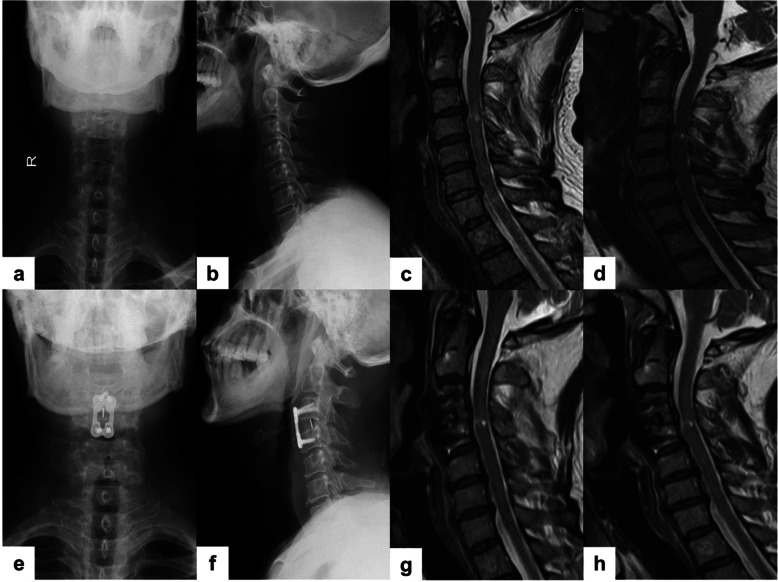
**a**, **b** X-ray showing no fracture and dislocation of the cervical spine. **c** MRI revealing long spinal cord high signal in acute phase. **d** Re-examined MRI showing that the high signal of the spinal cord was shorter than before. **e**, **f** Postoperative X-ray revealing that the internal fixation position was appropriate. **g** Postoperative MRI showing that the high signal of the spinal cord was shortened compared with preoperative. **h** Recent follow-up MRI revealing spinal cord hyperintensity limitation and signal intensity recedes

Two patients in group B showed spinal cord abnormal signals on the first MRI, and later the abnormal signals became shorter and limited to a single segment after re-examination (Fig. 2). MRI examination was performed immediately after admission of 2 patients in this group. MRI was re-examined before the surgery (surgery within 3–7 days) and compared with the first MRI, which showed that the high signal segment was shortened.

**Fig. 3:**
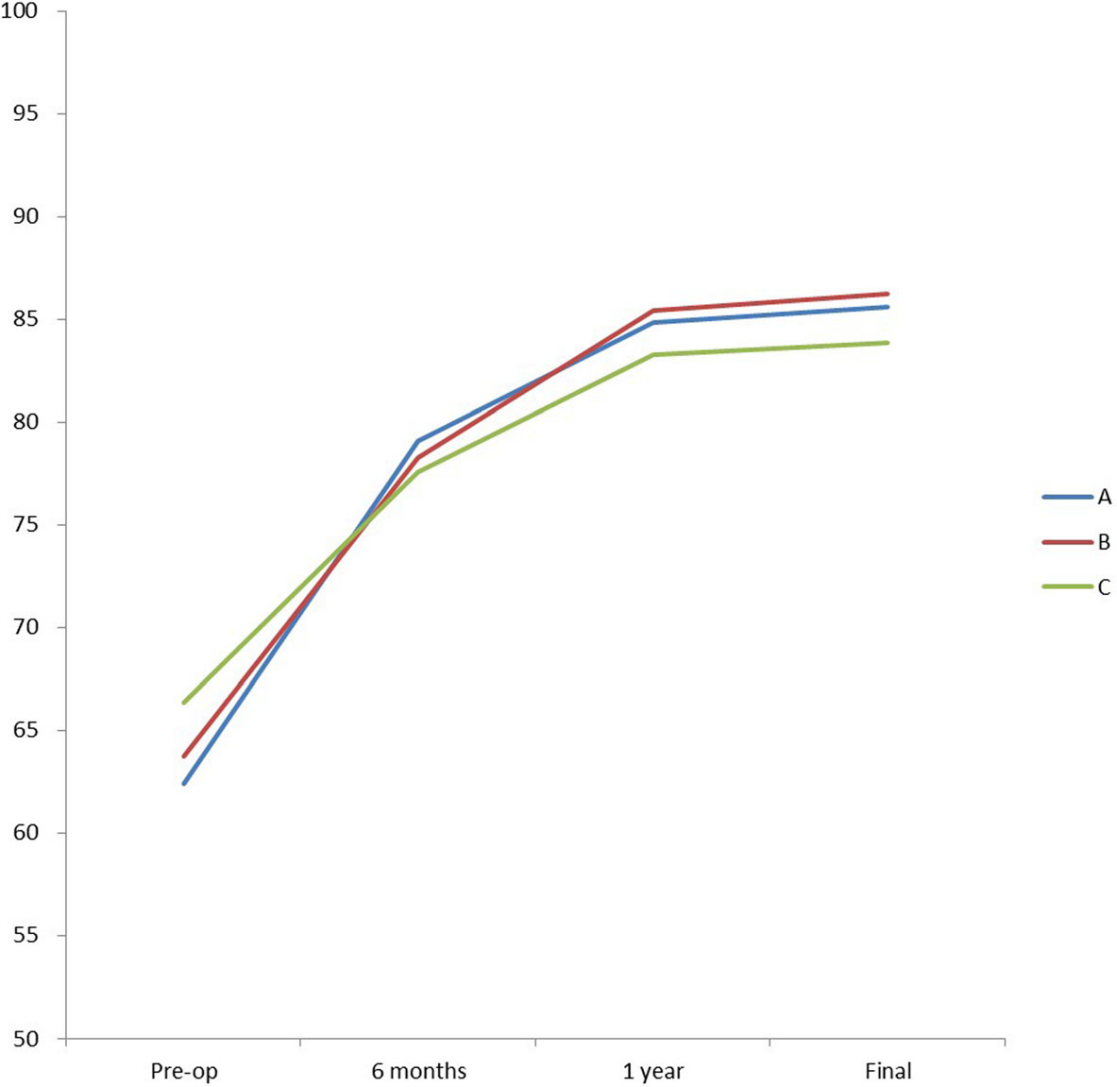
The trend of neurological status improvement among 3 groups. The ASIA sports score proved quick recovery in the early stage and slow recovery in the later stage

Through follow-up, it was found that the ASIA sports score had quickly recovered in the early stage, slower in the later stage, and in the final stage, the score did not increase significantly 1-year post-surgery (Fig. 3). The final follow-up showed that group B had better neurological function scores than the other 2 groups. Neurological function was no longer restored after 1 year of surgery compared to the final follow-up. Six months after the surgery, the recovery of ASIA sports score significantly slowed down. Image analysis showed that patients who completed surgery within 3 days had the fastest initial recovery (6-month follow-up), but patients who had completed surgery within 3–7 days had better neurological recovery with long-term follow-up (Fig. 3).

## Discussion

The mechanism of SCIWORA in adults has been controversial. A large number of studies believe that cervical spinal stenosis before trauma, such as cervical disc herniation, ossification of posterior longitudinal ligament, and calcification of ligamentum flavum, is closely related to the mechanism of SCIWORA. Patients with soft tissue injuries, such as anterior longitudinal ligament or ligamentum flavum injury, show less ability to improve. The external force reduces the storage capacity of the cervical spinal canal and compresses the spinal cord, which is consistent in the MRI findings of patients after injury [8–12]. MRI was carried out for 2 patients immediately after admission and underwent surgery within 3 days. MRI at final follow-up showed that abnormal signals still existed, which was not completely consistent with the first MRI. The results of MRI in SCIWORA within 3 days were incomplete, and delayed MRI was more significant. Thin section and high magnetic field MRI examinations can reveal lesions that cannot be monitored with routine MRI scans [13]. Samsani et al. [14] believed that after spinal injury, paravertebral venous plexus was abnormal, veins were tortuous and irritated, and paravertebral venous backflow was stagnant, which explained that the SCI segment was higher than the compression segment shown in MRI. The hemodynamics of SCI suggested that compressions could cause spinal cord vascular obstruction and thus alter the hemodynamics of the spinal cord. Instantaneous external force causes vascular injury of the nutrition spinal cord, bleeding, and edema, which aggravates the vascular injury again, forming a vicious cycle. However, the instability of SCIWORA had not been given enough attention, and cervical dynamic position X-ray should be executed with caution [11]. The diagnosis of SCIWORA in adults depends on plain radiographs and CT. As an important auxiliary means for the diagnosis of such injuries, MRI is becoming more important and is being recommended for routine review of the cervical spine [15]. MRI can detect characteristic pathomorphological soft tissue changes in patients with SCIWORA, including spinal cord hematoma, transverse section, herniated disc injury, spinal cord edema, and compression. Therefore, MRI has become a golden standard for diagnosing the SCI. The prognosis of SCIWORA also depends on the degree of initial neurological impairment and SCI revealed by MRI. Machino et al. [16] have demonstrated that even in the acute phase of MRI examination, SCIWORA patients with larger lesions were more likely to have poor neurological prognosis.

At present, conventional treatment methods include conservative and surgical treatments, and the latter is mainly based on anterior approach because the degree of such injuries is complicated and the majority of them are intervertebral disc injuries. Surgical treatment can significantly improve the prognosis of SCIWORA patients with extraneural or extra-intraneural MRI abnormalities. The surgery can be beneficial for both the long-term and short-term recovery of SCI. Wang et al. confirmed that SCIWORA was not associated with the destruction of adjacent vertebral bodies and endplates [17]. Compared with other surgical methods, anterior cervical surgery is simple, direct decompression, and can completely restore the physiological curvature of the spine, with fewer fusion segments and in a higher fusion rate, which can reduce the incidence of postoperative neck pain [18–20]. Posterior surgery was not recommended unless the patient had severe total cervical spinal stenosis. With the development of imaging and surgical techniques, surgical treatment has gradually become the mainstream method to treat SCIs, yet conservative treatment is still irreplaceable [21]. Mazaki et al. [22] treated the incomplete injury SCIWORA with conservative treatment and had a good prognosis. However, the long-term prognosis of conservative treatment for cervical spine instability is not good enough. Long-term follow-up showed that some patients were at enormous risk of secondary injury, with more severe post-injury symptoms than the first injury and poor prognosis [23]. Therefore, blind conservative treatment is also not recommended. Dynamic X-ray should be performed with caution and should be stopped as soon as the original symptoms become worse or new ones appear. Dynamic X-ray examination is not recommended for patients with definite injuries shown on MRI. Patients with negative MRI and positive flexion and extension X-ray are recommended to wear a neck collar for fixation. Re-examination will be conducted after 3 months. If cervical spine is still unstable, surgery is recommended to prevent secondary injury. Conservative treatment is recommended only when MRI, flexion, and extension X-ray are negative.

Fehlings et al. [24] suggested that early treatment should be taken for neurological injuries, and treatment commenced within 24 h could significantly improve the prognosis of the nervous system. The factors for early surgery are heavily dependent on MRI results and clinical manifestations [25, 26]. However, the abnormal signals of the spinal cord shown by early MRI cannot be used as an evaluation index of prognosis, and in some cases, it may be even missed, affecting the formulation of surgical plans. Re-examination should be done in a few days if results of early MRI are not satisfying [27–29]. Acute and delayed MRI findings are not identical, though edema can occur in the acute phase of SCI, such as high-intensity changes in T2. MRI findings are the result of long-term degenerative changes in the spinal cord. SCIWORA caused by degenerative spinal stenosis, with or without symptoms, will affect the changes of spinal cord signals on MRI. Delayed MRI examination can more accurately reflect the severity of clinical symptoms [27]. Studies have reported that X-ray, CT, and MRI are negative in some patients after SCI, which is called “real SCIWORA” [30, 31]. Spinal cord concussion and “real SCIWORA” are difficult to distinguish on MRI, but symptoms of spinal cord concussion generally disappear within 3 days, and neurological symptoms that persist after 3 days often indicate real injury [32]. Liu et al. [33] reported that the initial MRI of 2 patients was normal, and the re-examination after 72 h found abnormal manifestations, and finally, surgical intervention was carried out. Compared to the first MRI, if the abnormal MRI signal becomes shorter and the range is limited, further observation can be considered. Surgery can be performed after the spinal cord function is in the “plateau,” and MRI shows obvious compression. Prolonged compression of the spinal cord or nerve can cause irreversible damage. However, there is currently no relevant research on the “plateau” [34]. Two patients in group B underwent acute phase observation, and the final operative decompression and fixation segment were shorter than the damaged segment revealed in the first MRI, which may be related to the neglected spinal cord concussion or spinal cord edema (Fig. 2). Sagittal fluid-sensitive sequences that can identify edema (on the basis of a high T2 signal) and hemorrhage (on the basis of a low T2 signal) have been shown to have the most significant value in cases of SCIWORA [35, 36]. The prognosis of spinal cord edema is better than that of spinal cord hemorrhage.

This study had several limitations such as the small study sample size and the performance of the study in one center without control. Some studies had suggested that nerve recovery in patients undergoing surgery within 3–7 days was tremendous [37], but there are only a few literature reports on the observation of surgical efficacy beyond 7 days. In the future, a much larger sample size from many centers, controlled, and randomized studies will be required to help standardize the best procedure for the surgical treatment of SCIWORA.

## Conclusion

Treatment of patients with SCIWORA should be determined individually. Patients with clear MRI evidence of epidural lesions, including compression of the spinal cord, ligament damage and instability, and worsening or no improvement of neurological lesions, should be preferred for surgery. The timing of dynamic X-ray and MRI is very important, and their corresponding results directly affect the treatment options. Although the prognosis of all patients improved significantly within 7 days of surgery, the incidence of complications in premature surgery increased, and the surgical decompression range was affected. Early MRI examinations in a SCI within 3 days are difficult to fully find the signal, which may lead to misdiagnosis or missed diagnosis. The optimal timing of surgical treatment is 3–7 days after injury, which can significantly improve the short-term and long-term follow-up effects. Meanwhile, spinal cord concussion is excluded, which can lead to spinal cord decompression errors. Surgery performed after longer duration from the time injury worsens the prognosis of SCIWORA surgery.

## Data Availability

The datasets used and/or analyzed during the present study are available from the corresponding author on reasonable request.

## Abbreviations

SCIWORA: Spinal cord injury without radiographic abnormality
SCI: Spinal cord injury
ASIA: American Spinal Injury Association
AIS: ASIA Impairment Scale
CT: Computed tomography
MRI: Magnetic resonance imaging
ACDF: Anterior cervical discectomy with interbody fusion
ACCF: Anterior cervical corpectomy and fusion

## References

[1] Pang D, Pollack IF (1989). Spinal cord injury without radiographic abnormality in children--the SCIWORA syndrome. J Trauma.

[2] Hirsh LF, Duarte L, Wolfson EH (1993). Thoracic spinal cord injury without spine fracture in an adult: case report and literature review. Surg Neurol..

[3] Nagoshi N, Tetreault L, Nakashima H, Nouri A, Fehlings MG (2017). Return to play in athletes with spinal cord concussion: a systematic literature review. Spine J.

[4] Imajo Y, Hiiragi I, Kato Y, Taguchi T (2009). Use of the finite element method to study the mechanism of spinal cord injury without radiological abnormality in the cervical spine. Spine..

[5] Panjabi MM, Miura T, Cripton PA, Wang JL, Nain AS, DuBois C (2001). Development of a system for in vitro neck muscle force replication in whole cervical spine experiments. Spine..

[6] Song J, Mizuno J, Inoue T, Nakagawa H (2006). Clinical evaluation of traumatic central cord syndrome: emphasis on clinical significance of prevertebral hyperintensity, cord compression, and intramedullary high-signal intensity on magnetic resonance imaging. Surg Neurol..

[7] American Spinal Injury Association. International standards for neurological classification of spinal cord injury. http://asia-spinalinjury.org/wp-content/uploads/2016/02/International_Stds_Diagram_Worksheet.pdf. Accessed: July 8, 2016.

[8] Roe JP, Taylor TK, Edmunds IA, Cumming RG, Ruff SJ, Plunkett-Cole MD (2003). Spinal and spinal cord injuries in horse riding: the New South Wales experience 1976-1996. ANZ J Surg..

[9] Bernhardt M, Hynes RA, Blume HW, White AA (1993). Cervical spondylotic myelopathy. J Bone Joint Surg Am.

[10] Boese CK, Lechler P (2013). Spinal cord injury without radiologic abnormalities in adults: a systematic review. J Trauma Acute Care Surg..

[11] Kothari P, Freeman B, Grevitt M, Kerslake R (2000). Injury to the spinal cord without radiological abnormality (SCIWORA) in adults. J Bone Joint Surg Br.

[12] Bhatoe HS (2000). Cervical spinal cord injury without radiological abnormality in adults. Neurol India.

[13] Asan Z (2018). Spinal cord injury without radiological abnormality in adults: clinical and radiological discordance. World Neurosurg.

[14] Samsani SR, Calthorpe D, Geutjens G (2003). Thoracic spinal cord injury without radiographic abnormality in a skeletally mature patient: a case report. Spine..

[15] Goldberg AL, Rothfus WE, Deeb ZL, Daffner RH, Lupetin AR, Wilberger JE (1988). The impact of magnetic resonance on the diagnostic evaluation of acute cervicothoracic spinal trauma. Skeletal Radiol..

[16] Machino M, Yukawa Y, Ito K, Nakashima H, Kanbara S, Morita D (2011). Can magnetic resonance imaging reflect the prognosis in patients of cervical spinal cord injury without radiographic abnormality?. Spine..

[17] Huang SL, Yan HW, Wang KZ (2013). Use of Fidji cervical cage in the treatment of cervical spinal cord injury without radiographic abnormality. BioMed Res Int.

[18] Kabir SM, Alabi J, Rezajooi K, Casey AT (2010). Anterior cervical corpectomy: review and comparison of results using titanium mesh cages and carbon fibre reinforced polymer cages. Br J Neurosurg.

[19] Dvorak MF, Fisher CG, Aarabi B, Harris MB, Hurbert RJ, Rampersaud YR (2007). Clinical outcomes of 90 isolated unilateral facet fractures, subluxations, and dislocations treated surgically and nonoperatively. Spine..

[20] Kalayci M, Cagavi F, Acikgoz B (2004). Unilateral cervical facet fracture: presentation of two cases and literature review. Spinal Cord..

[21] Lauweryns P (2010). Role of conservative treatment of cervical spine injuries. Eur Spine J.

[22] Mazaki T, Ito Y, Sugimoto Y, Koshimune K, Tanaka M, Ozaki T (2013). Does laminoplasty really improve neurological status in patients with cervical spinal cord injury without bone and disc injury? A prospective study about neurological recovery and early complications. Arch Orthop Trauma Surg.

[23] Bosch PP, Vogt MT, Ward WT (2002). Pediatric spinal cord injury without radiographic abnormality (SCIWORA): the absence of occult instability and lack of indication for bracing. Spine..

[24] Fehlings MG, Vaccaro A, Wilson JR, Singh A, Cadotte DW, Harrop JS (2012). Early versus delayed decompression for traumatic cervical spinal cord injury: results of the Surgical Timing in Acute Spinal Cord Injury Study (STASCIS). PloS One.

[25] Mohanty SP, Bhat NS, Singh KA, Bhushan M (2013). Cervical spinal cord injuries without radiographic evidence of trauma: a prospective study. Spinal Cord..

[26] Boese CK, Lechler P (2014). Cervical spinal cord injuries without radiographic evidence of trauma: a prospective study. Spinal Cord..

[27] Ouchida J, Yukawa Y, Ito K, Katayama Y, Matsumoto T, Machino M (2016). Delayed magnetic resonance imaging in patients with cervical spinal cord injury without radiographic abnormality. Spine..

[28] Hayashi K, Yone K, Ito H, Yanase M, Sakou T (1995). MRI findings in patients with a cervical spinal cord injury who do not show radiographic evidence of a fracture or dislocation. Paraplegia..

[29] Shimada K, Tokioka T (1995). Sequential MRI studies in patients with cervical cord injury but without bony injury. Paraplegia..

[30] Dreizin D, Kim W, Kim JS, Boscak AR, Bodanapally UK, Munera F (2015). Will the real SCIWORA please stand up? Exploring clinicoradiologic mismatch in closed spinal cord injuries. AJR Am J Roentgenol.

[31] Yucesoy K, Yuksel KZ (2008). SCIWORA in MRI era. Clin Neurol Neurosurg.

[32] Asan Z (2018). Spinal Concussion in Adults: Transient neuropraxia of spinal cord exposed to vertical forces. World Neurosurg.

[33] Liu Q, Liu Q, Zhao J, Yu H, Ma X, Wang L (2015). Early MRI finding in adult spinal cord injury without radiologic abnormalities does not correlate with the neurological outcome: a retrospective study. Spinal Cord..

[34] Saruhashi Y, Hukuda S, Katsuura A, Asajima S, Omura K (1998). Clinical outcomes of cervical spinal cord injuries without radiographic evidence of trauma. Spinal Cord..

[35] Ajani AE, Cooper DJ, Scheinkestel CD, Laidlaw J, Tuxen DV (1998). Optimal assessment of cervical spine trauma in critically ill patients: a prospective evaluation. Anaesth Intensive Care.

[36] Bozzo A, Marcoux J, Radhakrishna M, Pelletier J, Goulet B (2011). The role of magnetic resonance imaging in the management of acute spinal cord injury. J Neurotrauma.

[37] Sapkas GS, Papadakis SA (2007). Neurological outcome following early versus delayed lower cervical spine surgery. J Orthop Surg (Hong Kong)..

